# Redistribution of heart failure as the cause of death: the Atherosclerosis Risk in Communities Study

**DOI:** 10.1186/1478-7954-12-10

**Published:** 2014-04-10

**Authors:** Michelle L Snyder, Shelly-Ann Love, Paul D Sorlie, Wayne D Rosamond, Carmen Antini, Patricia A Metcalf, Shakia Hardy, Chirayath M Suchindran, Eyal Shahar, Gerardo Heiss

**Affiliations:** 1 Department of Epidemiology, Gillings School of Public Health, University of North Carolina at Chapel Hill, 137 E. Franklin St., Suite 306, Chapel Hill, NC 27514, USA; 2 The National Heart, Lung, and Blood Institute (NHLBI), National Institutes of Health (NIH), Two Rockledge Center, Suite 10018, 6701 Rockledge Dr. MSC 7936, Bethesda, Maryland 20892, USA; 3 Department of Epidemiology, School of Public Health, University of Chile, Independencia 939, Independencia, Santiago 8380453, Chile; 4 Department of Statistics, University of Auckland, Private Bag 9201, Auckland 1142, New Zealand; 5 Department of Biostatistics, Gillings School of Public Health, University of North Carolina at Chapel Hill, 3103-A McGavran-Greenberg, 135 Dauer Drive, Chapel Hill, NC 27599, USA; 6 Division of Epidemiology and Biostatistics, Mel and Enid Zuckerman College of Public Health, The University of Arizona, 1295 N. Martin Ave, Tucson, AZ 85724, USA

**Keywords:** Cause of death, Coronary heart disease, Death certificates, Heart failure, Mortality, Vital statistics, Ill-defined causes of death

## Abstract

**Background:**

Heart failure is sometimes incorrectly listed as the underlying cause of death (UCD) on death certificates, thus compromising the accuracy and comparability of mortality statistics. Statistical redistribution of the UCD has been used to examine the effect of misclassification of the UCD attributed to heart failure, but sex- and race-specific redistribution of deaths on coronary heart disease (CHD) mortality in the United States has not been examined.

**Methods:**

We used coarsened exact matching to infer the UCD of vital records with heart failure as the UCD from 1999 to 2010 for decedents 55 years old and older from states encompassing regions under surveillance by the Atherosclerosis Risk in Communities (ARIC) Study (Maryland, Minnesota, Mississippi, and North Carolina). Records with heart failure as the UCD were matched on decedent characteristics (five-year age groups, sex, race, education, year of death, and state) to records with heart failure listed among the multiple causes of death. Each heart failure death was then redistributed to plausible UCDs proportional to the frequency among matched records.

**Results:**

After redistribution the proportion of deaths increased for CHD, chronic obstructive pulmonary disease, diabetes, hypertensive heart disease, and cardiomyopathy, *P* < 0.001. The percent increase in CHD mortality after redistribution was the highest in Mississippi (12%) and lowest in Maryland (1.6%), with variations by year, race, and sex. Redistribution proportions for CHD were similar to CHD death classification by a panel of expert reviewers in the ARIC study.

**Conclusions:**

Redistribution of ill-defined UCD would improve the accuracy and comparability of mortality statistics used to allocate public health resources and monitor mortality trends.

## Background

Accurate mortality statistics are critical for determining the burden of disease and evaluating mortality trends, but ill-defined causes of death such as heart failure are sometimes listed as the underlying cause of death (UCD) [1,2]. Heart failure is considered a mediator between death and diseases, thus it represents a mode of death most commonly attributable to hypertension, coronary heart disease (CHD), valve diseases, diabetes, or cardiomyopathy [3]. Cause of death coding instructions from the International Classification of Diseases Tenth Revision (ICD-10) state that other plausible heart conditions should be used as the UCD in place of heart failure. Reporting heart failure as the UCD most often occurs when the etiology of heart failure cannot be determined or training of the certifying physician [4] or the nosologist is inadequate.

Efforts to improve the comparability and accuracy of cause of death statistics call for redistributing UCDs attributed to ill-defined codes [1,2,5,6]. Medical record review by panels of experts or autopsy studies are the gold standard methods to determine the UCD. These methods are costly and laborious, and thus often limited to samples of available records [7-9] and to cohort settings [10,11]. Practical ways to redistribute ill-defined UCDs include statistical approaches such as multinomial logistic regression [2] that require preselecting underlying causes of death that heart failure deaths will be redistributed to [5] or use algorithms to redistribute heart failure deaths to a target list of UCDs [12].

Coarsened exact matching is a novel, validated approach to redistribute heart failure deaths [6]. In coarsened exact matching, records listing heart failure as the UCD are matched on selected variables to records listing heart failure as a multiple causes of death. Each heart failure death is then allocated to a plausible UCD proportional to the frequency of the underlying causes of death among the matched records.

Deaths attributed to heart failure are predominantly redistributed to CHD in the United States and most industrialized countries [1,5,6,12]. However, the effect of statistical redistribution of heart failure deaths on sex- and race-specific temporal trends in CHD mortality in the United States has not been examined. We report on the effect of redistributing heart failure deaths using coarsened exact matching on sex- and race-specific temporal trends in CHD mortality and compare coarsened exact matching to high-quality CHD cause of death ascertainment from the population-based surveillance study of United States communities, the Atherosclerosis Risk in Communities (ARIC) Study.

## Methods

### Vital records

We examined vital records from decedents ≥55 years old for years 1999–2010 from states encompassing regions under epidemiologic surveillance by the ARIC study (Maryland, Minnesota, Mississippi, and North Carolina). We excluded decedents <55 years old (n = 8) because heart failure before age 55 typically reflects etiologies and coding practices that differ from those among older adults in industrialized countries. Multiple cause of death coding was provided by the 57 vital statistics jurisdictions through the National Center for Health Statistics Vital Statistics Cooperative Program. We obtained mid-year populations for 1999–2009 from the Center for Disease Control’s CDC Wonder and obtained the 2010 mid-year population from the United States 2010 Census. Heart failure was defined by ICD-10 codes for congestive heart failure (I50.0), left ventricular failure (I50.1), and heart failure, unspecified (I50.9).

**Figure 1:**
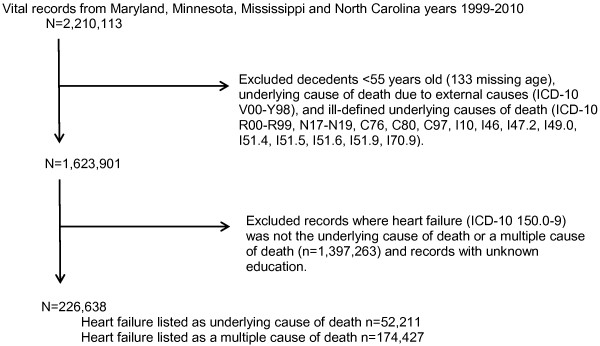
Flow diagram of exclusions.

Before matching we eliminated deaths due to external causes, ICD-10 codes V00-Y98, and deaths due to ill-defined UCD on the basis that they were not plausible as the UCD of a heart failure death (Figure 1). Each record with heart failure listed as the UCD was matched to records listing heart failure among the multiple causes of death by coarsened exact matching. The records were matched by five-year age groups (55 to 94 years old, and then 95+ years old), sex, race (Caucasian, African American, and other), education (less than high school, high school, and college and above), year of death, and state. Each heart failure death was then redistributed proportional to the frequency of the UCD of the matched records. For example, if a heart failure record matched to six CHD deaths, three cardiomyopathy deaths, and one diabetes death, the UCD for that heart failure record was redistributed as 0.6 CHD, 0.3 cardiomyopathy, and 0.1 diabetes death.

For the subsequent analysis, we grouped the following UCD as follows: CHD (I20-I25), chronic obstructive pulmonary disease (COPD; J40-J44), diabetes (E10-E14), hypertensive heart and kidney disease (I11-I13), cardiomyopathy (I42-I43), cerebrovascular disease (I60-I69), other cardiovascular diseases (balance of I00-I99), lower respiratory infections (J10-J18, J20-J22), cancers (C00-C97), digestive diseases (K20-K92), dementia (F01, F03), Alzheimer's disease (G30), and other diseases (remaining UCD).

### ARIC community surveillance

The National Heart, Lung, and Blood Institute-supported ARIC Community Surveillance Study includes annual sampling and validation of hospital discharge codes for myocardial infarction, heart failure, and in-hospital and out-of-hospital CHD mortality among residents 35 to 74 years old since 1987 from Forsyth County, North Carolina; the city of Jackson, Mississippi; suburbs of Minneapolis, Minnesota; and Washington County, Maryland. We included records with the UCD of heart failure for years 1999 to 2010 for decedents ≥55 years old from the four ARIC study areas. Detailed methods have been published [13,14] but briefly, deaths were sampled from each area stratified on sex, age, residence, year, and targeted ICD-10 codes related to CHD (ICD-10 codes E10-14, I10-11, I21-25, I46-51, I70, I79, J81, J96, R96, R98-99). Corresponding death certificates were obtained from state vital records with the UCD assigned by the Automated Classification of Medical Entities. Fatal events were characterized by contacting physicians and family members. Two members of a panel of physicians independently reviewed each out-of-hospital death and deaths with the UCD attributed to heart failure and assigned a final diagnosis using standardized criteria [15]. Disagreements between reviewers were adjudicated for a final CHD classification. The study was approved by the following institutional review boards: University of North Carolina at Chapel Hill, Wake Forest Baptist Medical Center, University of Mississippi Medical Center, University of Minnesota, and Johns Hopkins University.

For the purpose of this analysis, deaths were classified as a CHD death (definite myocardial infarction, and definite and possible fatal CHD), a non-CHD death, and an unclassifiable death in which the panel reviewers could not confirm or rule out a CHD death. We also considered a reviewer’s preferred classification in which the clinical judgment of the reviewer was used to complement the standardized ARIC classification. The preferred classification allowed the physician reviewer to disagree with the ARIC algorithm for a CHD death based on information in the medical record and his/her expert opinion.

### Statistical methods

We applied coarsened exact matching as proposed by Stevens et al. [6] implemented in the Stata procedure CEM [16]. Redistribution proportions among records with heart failure as the UCD were evaluated by state, race, and sex. Proportions of the UCD among all vital records before and after redistributing heart failure deaths were stratified by sex and race. Differences in pre- and post-redistribution proportions were tested with the chi-square test.

We calculated CHD mortality per 100,000 population using the direct method of standardization to the 2000 United States standard population. The percent increase in CHD mortality after redistributing heart failure deaths was calculated as [(*post-redistribution morality rate* − *pre-distribution morality rate*)/*pre-distribution mortality rate*] × 100. We graphed CHD mortality and percent increase in CHD mortality by region, sex, and race to evaluate trends over time. We suppressed data for “other” race and African Americans in Minnesota when calculating CHD mortality rates due to the small number of records.

Frequencies of the CHD classification of deaths in ARIC were estimated by the inverse of the sampling fractions and rounded to the nearest whole number. SAS, version 9.2 (SAS Institute, Inc., Cary, North Carolina) was used for all analyses except for coarsened exact matching that was conducted with Stata, version 12.1 (Stata Corporation, College Station, Texas). All *P* values were two-sided with statistical significance of *P* <0.05.

## Results

Heart failure was listed as the UCD in 3.2% (n = 52,211) of all vital records, with 2.0% in Maryland, 3.7% in Minnesota, 6.4% in Mississippi, and 2.6% in North Carolina (Additional file 1: Table S1). Among records with heart failure as the UCD, 46.7% of the decedents were male and 53.3% were female, and 84.6% of the decedents were Caucasian compared to 14.8% African American and 0.6% other race (Additional file 1: Table S2). However, 25.3% of the decedents in Mississippi were African American compared to 0.9% in Minnesota.

Among records with heart failure as the UCD, coarsened exact matching redistributed 37.1% of the deaths to CHD, 10.8% to other cardiovascular diseases, 9.9% to other, 8.0% to COPD, 6.7% to cancers, 4.9% to diabetes, 4.5% to hypertensive heart and kidney disease, 4.4% to cerebrovascular disease, and a lower proportion to each of the other UCD groups (Table 1). Redistribution proportions varied slightly across states for CHD (ranging from 33.4% in Minnesota to 39.1% in Mississippi) and hypertensive heart and kidney disease (ranging from 3.3% in Minnesota to 6.7% in Mississippi). After redistributing heart failure deaths, the proportion of deaths among all vital records significantly increased for CHD, COPD, diabetes, hypertensive heart and kidney disease, cardiomyopathy, and other cardiovascular diseases in Caucasians and females (Table 2). However, only CHD, COPD, and other cardiovascular diseases significantly increased in African Americans and only CHD, COPD, cardiomyopathy, and other cardiovascular diseases significantly increased in males.

**Table 1:** Redistribution proportions among records with heart failure as the underlying cause of death by state, sex, and race

**Underlying cause of death**	**All records**	**Maryland**	**Minnesota**	**Mississippi**	**North Carolina**	**Male**	**Female**	**Caucasian**	**African American**
	**%**	**%**	**%**	**%**	**%**	**%**	**%**	**%**	**%**
Lower respiratory infections	3.3	3.1	3.1	3.5	3.2	3.2	3.3	3.4	2.6
Cancers	6.7	6.4	6.9	7.5	5.9	7.2	6.2	6.4	8.4
Diabetes	4.9	5.1	5.7	4.4	4.7	5.1	4.8	4.4	7.8
Coronary heart disease	37.1	39.0	33.4	39.1	37.4	39.3	35.2	37.9	32.6
Cerebrovascular disease	4.4	3.8	4.5	5.2	4.0	4.4	4.4	4.3	5.4
Hypertensive heart and kidney disease	4.5	4.2	3.3	6.7	3.8	3.9	5.1	3.8	8.7
Cardiomyopathy	3.3	3.5	1.9	3.8	3.8	3.6	3.1	2.9	5.4
Other cardiovascular diseases	10.8	10.8	14.4	6.8	11.6	9.9	11.7	11.4	7.5
Chronic obstructive pulmonary disease	8.0	7.4	7.9	8.3	8.1	8.0	8.0	8.5	4.9
Digestive diseases	2.3	2.2	2.5	2.2	2.3	2.3	2.4	2.3	2.2
Other	9.9	10.9	10.2	8.5	10.3	9.6	10.1	9.6	11.1
Dementia	2.7	2.3	4.0	1.8	2.8	1.9	3.5	2.9	2.0
Alzheimer’s disease	2.1	1.4	2.4	2.2	2.1	1.7	2.4	2.2	1.4

**Table 2:** Proportions of the underlying cause of death pre- and post-redistribution of heart failure deaths

**Underlying cause of death**	**By race**^**a**^				**By sex**			
	**Caucasian**		**African American**		**Male**		**Female**	
	**(n = 1,330,997)**		**(n = 275,023)**		**(n = 794,546)**		**(n = 829,355)**	
	**Pre%**	**Post%**	**Pre%**	**Post%**	**Pre%**	**Post%**	**Pre%**	**Post%**
Heart failure	3.3	0.0	2.8	0.0	3.1	0.0	3.4	0.0
Coronary heart disease	19.7	20.9^b^	19.9	20.8^b^	20.7	21.9^b^	18.8	19.9^b^
Chronic obstructive pulmonary disease	6.9	7.2^b^	3.2	3.4^b^	6.2	6.4^b^	6.3	6.5^b^
Diabetes	3.0	3.1^b^	5.7	5.9	3.6	3.7	3.3	3.5^b^
Hypertensive heart and kidney disease	1.5	1.6^b^	4.1	4.3	1.9	2.0	1.9	2.1^b^
Cardiomyopathy	1.1	1.2^b^	1.6	1.7	1.2	1.3^b^	1.1	1.2^b^
Cerebrovascular disease	7.8	7.9	8.3	8.4	7.6	7.8	8.1	8.2
Other cardiovascular diseases	4.7	5.1^b^	4.0	4.2^b^	4.4	4.7^b^	4.8	5.2^b^
Lower respiratory infections	3.0	3.1	2.5	2.6	2.9	3.0	2.9	3.0
Cancers	24.4	24.6	24.7	24.9	25.5	25.7	23.5	23.7
Digestive diseases	3.7	3.8	3.4	3.4	3.7	3.8	3.6	3.7
Dementia	4.5	4.5	3.1	3.1	3.2	3.3	5.1	5.2
Alzheimer’s disease	3.9	4.0	2.3	2.4	3.0	3.1	4.3	4.3
Other diseases	12.8	13.1^b^	14.5	14.8	13.1	13.4	13	13.4^b^

^a^Other race not shown (n = 17,881).

^b^*P*-value <0.05 from chi-square test.

**Figure 2:**
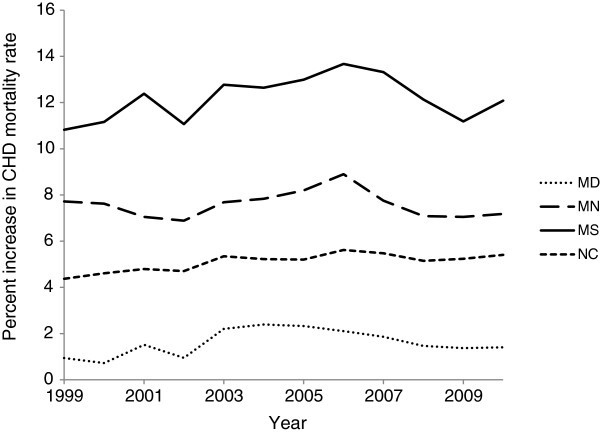
**Percent increase in age-adjusted mortality rate for coronary heart disease after redistribution of heart failure deaths by state.** Age-adjusted mortality rate is per 100,000 population among US adults ≥55 years, age-standardized to the 2000 US standard population. MD, Maryland; MN, Minnesota; MS, Mississippi; NC, North Carolina.

**Figure 3:**
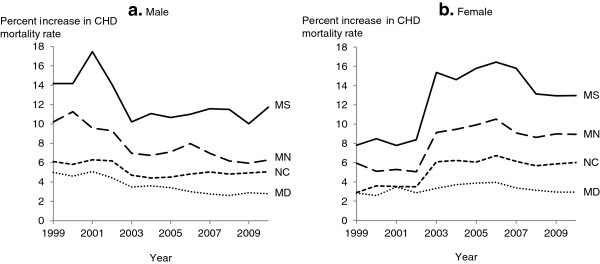
**Percent increase in age-adjusted mortality rate for coronary heart disease after redistribution of heart failure deaths by sex and state.** Age-adjusted mortality rate is per 100,000 population among US (**a**) male and (**b**) female adults ≥55 years, age-standardized to the 2000 US standard population. MD, Maryland; MN, Minnesota; MS, Mississippi; NC, North Carolina.

**Figure 4:**
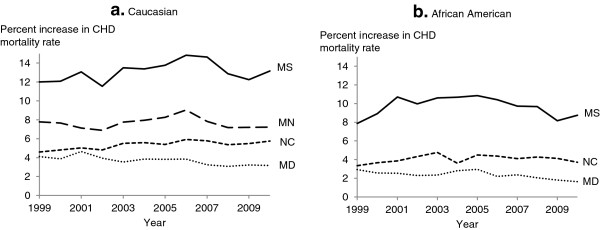
**Percent increase in age-adjusted mortality rate for coronary heart disease after redistribution of heart failure deaths by race and state.** Age-adjusted mortality rate is per 100,000 population among US (**a**) Caucasian and (**b**) African American adults ≥55 years, age-standardized to the 2000 US standard population. Data for African Americans in Minnesota were suppressed because of small numbers. MD, Maryland; MN, Minnesota; MS, Mississippi; NC, North Carolina.

The percent increase in CHD mortality after redistributing heart failure deaths varied by year and state, ranging from 0.7% in Maryland to 13.7% in Mississippi (Figure 2). The percent increase in CHD mortality was the lowest and had little variation over time in Maryland and was the highest and had a large variation over time in Mississippi. The percent increase in CHD following redistribution was higher among males than females in all states until around 2002, at which point females had a higher percent increase starting around 2009, except for Maryland (Figure 3). The percent increase in CHD mortality was higher among Caucasians compared to African Americans in Maryland, Mississippi, and North Carolina (Figure 4).

Post-redistribution mortality rates of CHD were consistently higher than pre-redistribution rates with comparable trends over time among all states (Additional file 1: Figure S1). CHD mortality was lower among females compared to males in all states (Additional file 1: Figure S2). There was a larger decrease in CHD mortality among males around 2001 to 2003 in all states except Minnesota, which was not seen among females. CHD mortality was the highest overall among African Americans in Maryland (Additional file 1: Figure S3).

In ARIC Community Surveillance regions, there were 660 records with heart failure listed as the UCD from 1999 to 2010. The majority of records were from Mississippi (39.7%) and North Carolina (34.5%; Additional file 1: Table S3). Based on the ARIC classification algorithm protocol, 22.3% of records with heart failure as the UCD were classified as a CHD death (24.7% among males, 19.6% among females, 22.4% among Caucasians, and 22.6% among African Americans; Additional file 1: Table S4). For the physician reviewer’s preferred classification, records with heart failure as the UCD were classified as a CHD death in 27.5% of all the records (31.5% among males, 22.8% among females, 29.8% among Caucasians, and 24.7% among African Americans). Jackson, Mississippi had the largest proportion of heart failure deaths classified as CHD deaths (28.1%) followed by Forsyth County, North Carolina (22.5%; Additional file 1: Table S5). There were few records with heart failure as the UCD in Minneapolis, Minnesota and Washington County, Maryland.

## Discussion

Misclassification of deaths due to ill-defined UCD remains a problem in national vital statistics. Prominent among the ill-defined UCD is heart failure, which was listed as the UCD in over 3% of all vital records from 1999 to 2010. On a national scale, this represents approximately 63,273 deaths in 2010 among United States decedents ≥55 years old. Deaths attributed to heart failure redistributed by coarsened exact matching were predominantly assigned to CHD, with variations in redistribution proportions by state, sex, and race. Similar redistribution patterns were seen for heart failure deaths classified by a physician reviewer panel following ARIC’s Community Surveillance protocol. These approaches reclassified 22% to 37% of deaths coded as heart failure to CHD deaths and classified more heart failure deaths as CHD in Mississippi and among males. CHD mortality increased by 0.7% to 13.7% among the four states after redistributing heart failure deaths, suggesting that without redistributing heart failure deaths, CHD mortality based on vital statistics is underestimated by 0.7% to 13.7% in the four states included.

As shown in our analysis and those of others [5,6,12], heart failure deaths are mainly redistributed to CHD in economically advanced, industrialized settings. Stevens et al. used coarsened exact matching and showed that heart failure deaths were primarily redistributed to CHD, COPD, diabetes, and hypertensive heart and kidney disease [6]. Using a regression-based approach, Ahern et al. also redistributed most heart failure deaths to CHD [12] although all heart failure deaths were redistributed to CHD among males, and 87% of the heart failure deaths were redistributed to CHD and 13% to COPD among females, which was not the case in the present study. An advantage of coarsened exact matching in this regard is that no *a priori* assumptions about the UCD target groups are necessary, and a heart failure death may be redistributed to several underlying causes of death. Stevens et al. reported that the overall percent increase in mortality for CHD after coarsened exact matching did not vary over time between 1999 to 2004 in the United States [6]. To this we add the hypothesized observation of state-level variation in the percent increase in CHD due to redistribution. Furthermore, the percent increase in CHD mortality after redistribution was higher in males compared to females from 1990 to 2002, whereas from 2002 to 2010 females had a higher percent increase. The larger decline in CHD mortality from 2001 to 2003 among males compared to females in our analysis may contribute to this pattern.

Misclassification of the UCD due to ill-defined codes has been reported to vary by location [2,17], the certifying physician [4], sex, race [18], and also with older age of the decedent [2,5]. Murray et al. reported that in-hospital deaths and deaths in counties with more cardiologists per capita were less likely assigned to heart failure or general atherosclerosis compared to CHD [2]. Misclassification of heart failure could also be due to differences in the training of nosologists by state and the use of commercial firms to perform the coding.

There is plausibility for heart failure deaths to be redistributed to CHD where the predominant underlying mechanism of heart failure is ischemic cardiomyopathy [19,20], and the majority of deaths coded as heart failure or other ill-defined cardiovascular UCD are redistributed to CHD when adjudicated by an expert panel [10]. We compared the statistical approach to redistributing heart failure deaths to a standardized and rigorous method of reclassification by a panel of physicians, based on reviews of medical records and surveys of the decedent’s physician and next of kin. The statistical approach redistributed a higher percentage of heart failure deaths to CHD than the panel of physicians, but the latter identified some records as unclassifiable. Both methods resulted in similar redistribution patterns and yielded comparable mortality estimates that reduce the impact of misclassification of the UCD in the medical certification of deaths.

Our study is limited by the lack of autopsy data to determine the true UCD, given that very few autopsies are conducted in the United States. Also to be considered, the method we used assigns deaths to plausible UCD and records could therefore be redistributed to different UCD to account for the uncertainty of the true UCD. Our analysis is based on vital records that may have inaccurate demographic information and cause of death coding; however, redistribution of heart failure offers a way to improve the misclassification of the UCD and comparability of mortality statistics. Lastly, this analysis only included four states in the United States to allow for comparison with an established community surveillance program. Even within this constrained framework, we were able to detect geographic variations in the redistribution of heart failure deaths both from vital records and the ARIC study. We also were able to compare temporal trends in CHD mortality by race and sex to previous studies that used redistribution of deaths due to ill-defined causes based on vital records prior to 2008 [2,5,6,12].

Efforts to improve vital statistics are not new. Other studies have shown feasible ways to calibrate mortality to account for ill-defined UCD [2,6,12,21], but these methods are not commonly used or cited. Considering that cause-specific mortality estimates for heart failure as the UCD are available on CDC Wonder, there is a clear need to inform public health researchers of the potential for misclassification of the UCD due to ill-defined causes such as heart failure when evaluating mortality statistics, as well as a need for practical tools to calibrate such data. Emphasis needs to be on improving the quality of medical records and the training of physicians on death certification and reporting multiple cause of death information to provide a more accurate estimation of the burden of disease [22], especially since diseases and conditions frequently co-exist. Despite the possibilities of inaccuracy and misclassification, the ICD system is used around the world to monitor diseases and for public health policies.

## Conclusions

In summary, coarsened exact matching offers a practical way to redistribute ill-defined UCD. Further research is needed to validate this method, but our results indicate that redistribution of deaths attributed to heart failure modifies cause-specific mortality estimates and may significantly impact mortality patterns for subgroups or regions prone to suboptimal certification of the UCD. Government agencies and researchers should calibrate vital statistics through reclassification of ill-defined UCD in order to improve the accuracy and comparability of mortality statistics, to the benefit of monitoring mortality trends, public health policies, and the allocation of public health resources.

## Abbreviations

ARIC: Atherosclerosis risk in communities study; CHD: Coronary heart disease; COPD: Chronic obstructive pulmonary disease; ICD-10: The international classification of disease tenth revision; UCD: Underlying causes of death.

## Competing interests

The authors declare that they have no competing interests.

## Authors’ contributions

All authors read and approved the final version of the manuscript and its submission to the journal. Specifically, MLS, SL, PDS, WDR, CA, PAM, SH, CMS, ES, and GH contributed to the concept and design of the study. MLS, SL, WDR, and GH acquired the data. MLS, SL, PAM, CMS, and GH contributed to the analysis and interpretation of the data, and PDS, WDR, CA, SH, and ES contributed to the interpretation of the data. MLS and GH led the drafting of the manuscript and SL, PDS, WDR, CA, PAM, SH, CMS, and ES reviewed and revised the manuscript.

## Supplementary Material

Additional file 1: Table S1 Characteristics of decedents, **Table S2.** Characteristics of decedents with heart failure listed as the UCD, **Figure S1.** Age-adjusted mortality rate of CHD pre- and postredistribution of heart failure deaths by state, **Figure S2.** Age-adjusted mortality rate of CHD pre- and postredistribution of heart failure deaths by sex and state, **Figure S3.** Age-adjusted mortality rate of CHD pre- and postredistribution of heart failure deaths by race and state, **Table S3.** ARIC community coronary heart disease surveillance decedent characteristics of records listing heart failure as the UCD, **Table S4.** ARIC community surveillance CHD classification of records listing heart failure as the UCD, **Table S5.** ARIC community surveillance CHD classification of records listing heart failure as the UCD by field center. Click here for file
