# Supplementary material for: Redistribution of heart failure as the cause of death: the Atherosclerosis Risk in Communities Study
Source: Popul Health Metr. 2014 Apr 10;12:10. doi: 10.1186/1478-7954-12-10 (PMC4113199; doi:10.1186/1478-7954-12-10)
Supplement: Additional file 1: Table S1 — Characteristics of decedents, Table S2. Characteristics of decedents with heart failure listed as the UCD, Figure S1. Age-adjusted mortality rate of CHD pre- and postredistribution of heart failure deaths by state, Figure S2. Age-adjusted mortality rate of CHD pre- and postredistribution of heart failure deaths by sex and state, Figure S3. Age-adjusted mortality rate of CHD pre- and postredistribution of heart failure deaths by race and state, Table S3. ARIC community coronary heart disease surveillance decedent characteristics of records listing heart failure as the UCD, Table S4. ARIC community surveillance CHD classification of records listing heart failure as the UCD, Table S5. ARIC community surveillance CHD classification of records listing heart failure as the UCD by field center. [file 1478-7954-12-10-S1.docx]

| **Table S1.** **Characteristics of decedents from 1999 to 2010^a^** | | | | | | | | | | | | | | | | | | | |
| --- | --- | --- | --- | --- | --- | --- | --- | --- | --- | --- | --- | --- | --- | --- | --- | --- | --- | --- | --- |
| **Characteristic** | | **All** | |  | **Maryland** | | |  | | **Minnesota** | | |  | **Mississippi** | |  | | **North Carolina** | |
|  |  | **No. (%)** | |  | **No. (%)** | | |  | | **No. (%)** | | |  | **No. (%)** | |  | | **No. (%)** | |
| Total records | | 1,623,901 (100) | |  | 395,419 (24.35) | | |  | | 342,603 (21.10) | | |  | 227,009 (13.98) | |  | | 658,870 (40.57) | |
| Heart failure as the underlying cause of death | | 52,211 (3.22) | |  | 7,737 (1.96) | | |  | | 12,808 (3.74) | | |  | 14,558 (6.41) | |  | | 17,108 (2.60) | |
| Heart failure as a multiple cause of death | | 174,427 (10.74) | |  | 37,086 (9.38) | | |  | | 40,441 (11.80) | | |  | 30,237 (13.32) | |  | | 66,663 (10.12) | |
| Sex | |  | |  |  | | |  | |  | | |  |  | |  | |  | |
| Male | | 794,546 (48.93) | |  | 192,717 (48.74) | | |  | | 167,648 (48.93) | | |  | 112,106 (49.38) | |  | | 322,075 (48.88) | |
| Female | | 829,355 (51.07) | |  | 202,702 (51.26) | | |  | | 174,955 (51.07) | | |  | 114,903 (50.62) | |  | | 336,795 (51.12) | |
| Age group | |  | |  |  | | |  | |  | | |  |  | |  | |  | |
| 55-59 | | 98,958 (6.09) | |  | 23,982 (6.06) | | |  | | 16,606 (4.85) | | |  | 16,044 (7.07) | |  | | 42,326 (6.42) | |
| 60-64 | | 121,892 (7.51) | |  | 29,091 (7.36) | | |  | | 20,145 (5.88) | | |  | 19,601 (8.63) | |  | | 53,055 (8.05) | |
| 65-69 | | 148,970 (9.17) | |  | 34,859 (8.82) | | |  | | 25,336 (7.40) | | |  | 23,479 (10.34) | |  | | 65,296 (9.91) | |
| 70-74 | | 190,258 (11.72) | |  | 44,985 (11.38) | | |  | | 34,422 (10.05) | | |  | 29,045 (12.79) | |  | | 81,806 (12.42) | |
| 75-79 | | 245,037 (15.09) | |  | 60,054 (15.19) | | |  | | 47,607 (13.90) | | |  | 34,807 (15.33) | |  | | 102,569 (15.57) | |
| 80-84 | | 285,928 (17.61) | |  | 71,334 (18.04) | | |  | | 61,327 (17.90) | | |  | 37,751 (16.63) | |  | | 115,516 (17.53) | |
| 85-89 | | 270,819 (16.68) | |  | 67,357 (17.03) | | |  | | 65,450 (19.10) | | |  | 33,870 (14.92) | |  | | 104,142 (15.81) | |
| 90-94 | | 178,777 (11.01) | |  | 43,514 (11.00) | | |  | | 47,751 (13.94) | | |  | 21,912 (9.65) | |  | | 65,600 (9.96) | |
| 95+ | | 83,262 (5.13) | |  | 20,243 (5.12) | | |  | | 23,959 (6.99) | | |  | 10,500 (4.63) | |  | | 28,560 (4.33) | |
| Race | |  | |  |  | | |  | |  | | |  |  | |  | |  | |
| Caucasian | | 1,330,997 (81.96) | |  | 301,975 (76.37) | | |  | | 333,419 (97.32) | | |  | 162,775 (71.70) | |  | | 532,828 (80.87) | |
| African American | | 275,023 (16.94) | |  | 87,788 (22.20) | | |  | | 4,518 (1.32) | | |  | 63,439 (27.95) | |  | | 119,278 (18.10) | |
| Other | | 17,881 (1.10) | |  | 5,656 (1.43) | | |  | | 4,666 (1.36) | | |  | 795 (0.35) | |  | | 6,764 (1.03) | |
| Education | |  | |  |  | | |  | |  | | |  |  | |  | |  | |
| Less than high school | | 630,554 (38.83) | |  | 124,802 (31.56) | | |  | | 110,189 (32.16) | | |  | 96,866 (42.67) | |  | | 298,697 (45.33) | |
| High school graduate | | 560,822 (34.54) | |  | 157,125 (39.74) | | |  | | 130,746 (38.16) | | |  | 79,394 (34.97) | |  | | 193,557 (29.38) | |
| College and above | | 432,525 (26.63) | |  | 113,492 (28.70) | | |  | | 101,668 (29.68) | | |  | 50,749 (22.36) | |  | | 166,616 (25.29) | |
| ^a^ Dataset after excluding external UCD and select UCD due to renal failure, essential hypertension and general unspecified atherosclerosis | | | | | | | | | | | | | | | | | | | |
| **Table S2. Characteristics of decedents with heart failure listed as the underlying cause of death from 1999 to 2010^a^** | | | | | | | | | | | | | | | | | |  |  |
| **Characteristic** | | **All** | | |  | **Maryland** | |  | | **Minnesota** |  | | **Mississippi** |  | | **North Carolina** | |  |  |
|  |  | **No. (%)** | | |  | **No. (%)** | |  | | **No. (%)** |  | | **No. (%)** |  | | **No. (%)** | |  |  |
| Total records | | 52,211 (100) | | |  | 7,737 (14.82) | |  | | 12,808 (24.53) |  | | 14,558 (27.88) |  | | 17,108 (32.77) | |  |  |
| Sex | |  | | |  |  | |  | |  |  | |  |  | |  | |  |  |
| Male | | 24,376 (46.69) | | |  | 3,775 (48.79) | |  | | 5,975 (46.65) |  | | 6,728 (46.22) |  | | 7,898 (46.17) | |  |  |
| Female | | 27,835 (53.31) | | |  | 3,962 (51.21) | |  | | 6,833 (53.35) |  | | 7,830 (53.78) |  | | 9,210 (53.83) | |  |  |
| Age group | |  | | |  |  | |  | |  |  | |  |  | |  | |  |  |
| 55-59 | | 1,009 (1.93) | | |  | 136 (1.76) | |  | | 100 (0.78) |  | | 391 (2.69) |  | | 382 (2.23) | |  |  |
| 60-64 | | 1,533 (2.94) | | |  | 203 (2.62) | |  | | 156 (1.22) |  | | 586 (4.03) |  | | 588 (3.44) | |  |  |
| 65-69 | | 2,212 (4.24) | | |  | 336 (4.34) | |  | | 275 (2.15) |  | | 777 (5.34) |  | | 824 (4.82) | |  |  |
| 70-74 | | 3,548 (6.80) | | |  | 483 (6.24) | |  | | 551 (4.30) |  | | 1,183 (8.13) |  | | 1,331 (7.78) | |  |  |
| 75-79 | | 6,096 (11.68) | | |  | 955 (12.34) | |  | | 1,117 (8.72) |  | | 1,885 (12.95) |  | | 2,139 (12.50) | |  |  |
| 80-84 | | 9,276 (17.77) | | |  | 1,411 (18.24) | |  | | 2,103 (16.42) |  | | 2,518 (17.30) |  | | 3,244 (18.96) | |  |  |
| 85-89 | | 12,108 (23.19) | | |  | 1,848 (23.89) | |  | | 3,248 (25.36) |  | | 3,178 (21.83) |  | | 3,834 (22.41) | |  |  |
| 90-94 | | 10,254 (19.64) | | |  | 1,484 (19.18) | |  | | 3,159 (24.66) |  | | 2,518 (17.30) |  | | 3,093 (18.08) | |  |  |
| 95+ | | 6,175 (11.83) | | |  | 881 (11.39) | |  | | 2,099 (16.39) |  | | 1,522 (10.45) |  | | 1,673 (9.78) | |  |  |
| Race | |  | | |  |  | |  | |  |  | |  |  | |  | |  |  |
| Caucasian | | 44,164 (84.59) | | |  | 6,391 (82.60) | |  | | 12,596 (98.34) |  | | 10,847 (74.51) |  | | 14,330 (83.76) | |  |  |
| African American | | 7,733 (14.81) | | |  | 1,270 (16.41) | |  | | 112 (0.87) |  | | 3,682 (25.29) |  | | 2,669 (15.60) | |  |  |
| Other | | 314 (0.60) | | |  | 76 (0.98) | |  | | 100 (0.78) |  | | 29 (0.20) |  | | 109 (0.64) | |  |  |
| Education | |  | | |  |  | |  | |  |  | |  |  | |  | |  |  |
| Less than high school | | 23,899 (45.77) | | |  | 2,749 (35.53) | |  | | 5,298 (41.36) |  | | 7,038 (48.34) |  | | 8,814 (51.52) | |  |  |
| High school graduate | | 16,406 (31.42) | | |  | 2,915 (37.68) | |  | | 4,350 (33.96) |  | | 4,704 (32.31) |  | | 4,437 (25.94) | |  |  |
| College and above | | 11,906 (22.80) | | |  | 2,073 (26.79) | |  | | 3,160 (24.67) |  | | 2,816 (19.34) |  | | 3,857 (22.55) | |  |  |
| ^a^ Dataset after excluding decedents <55 years old (133 with missing age) and records with an underlying cause of death due to external causes (ICD-10 V00-Y89) and ill-defined causes of death (ICD-10 R00-R99, N17-N19, C76, C80, C97, I10, I46, I472, I490, I514, I515, I516, I519, I709). | | | | | | | | | | | | | | | | | |  |  |

**Figure S1.** Age-adjusted mortality rate of coronary heart disease by state pre- and post-redistribution of heart failure deaths. Rates are among U.S adults ≥55 years old age-standardized to the 2000 U.S. standard population. MD, Maryland; MN, Minnesota; MS, Mississippi; NC, North Carolina.


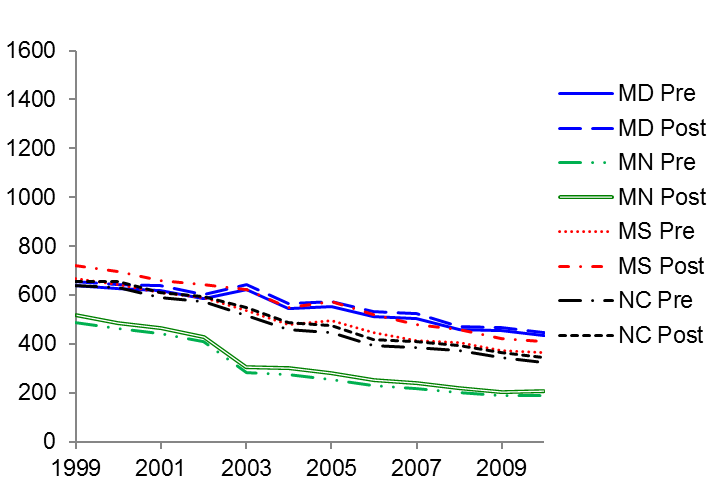

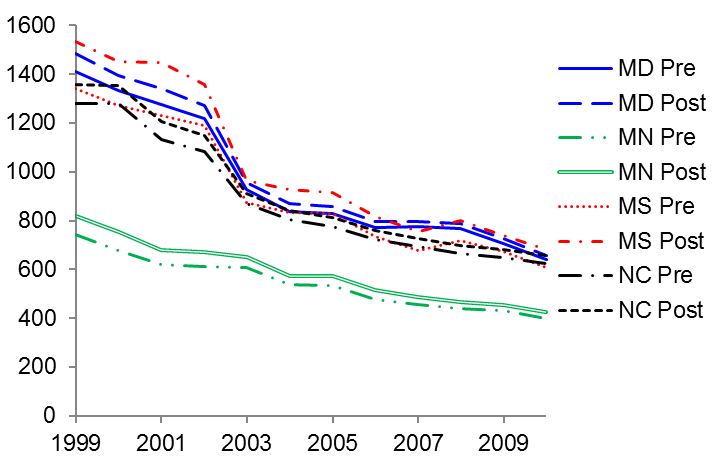


Rate per 100,000 population

Rate per 100,000 population

a. Male

b. Female

Year

Year

**Figure S2.** Age-adjusted mortality rate of coronary heart disease pre and post-redistribution of heart failure deaths by sex and state. Rates are per 100,000 population among US adults ≥55 years old age-adjusted and standardized to the 2000 US standard population. MD, Maryland; MN, Minnesota; MS, Mississippi; NC, North Carolina.


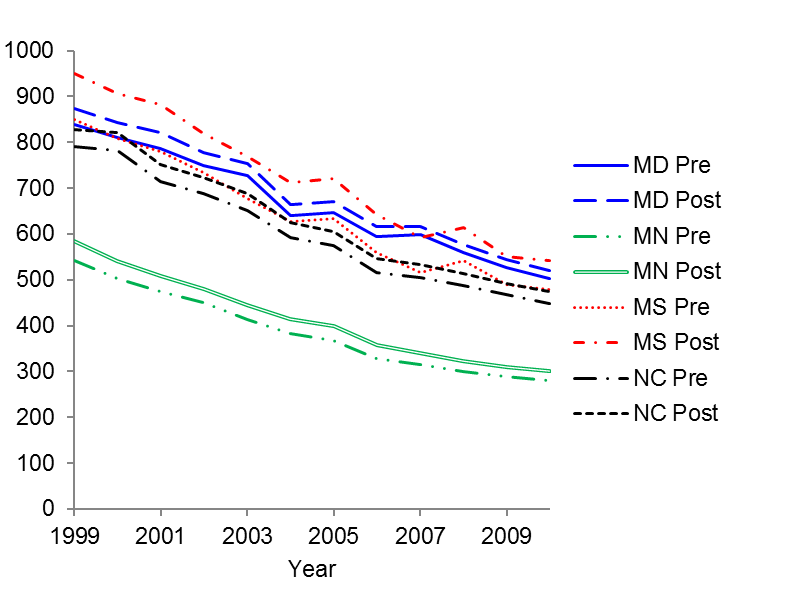

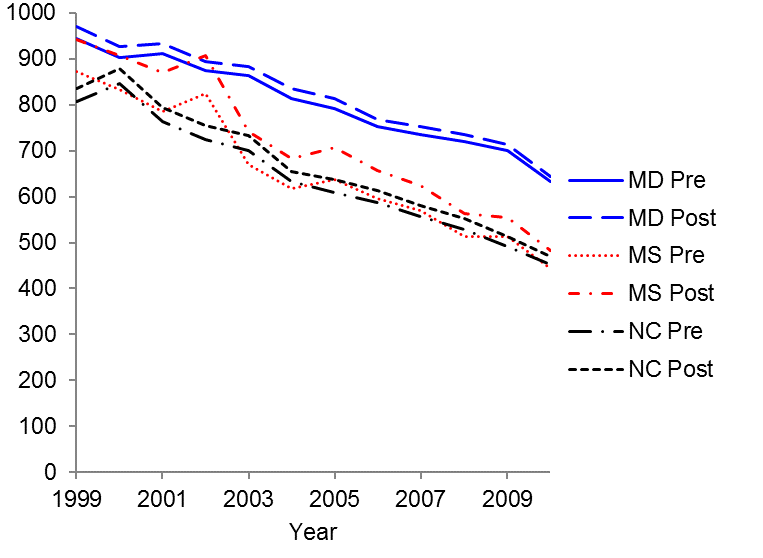


a. Caucasian

b. African American

Rate per 100,000 population

Rate per 100,000 population

**Figure S3.** Age-adjusted mortality rate of coronary heart disease pre and post-redistribution of heart failure deaths by race and state. Rates are per 100,000 population among US adults ≥55 years old age-adjusted and standardized to the 2000 US standard population. Data for African Americans in Minnesota were suppressed because numbers for were too small for a meaningful analysis. MD, Maryland; MN, Minnesota; MS, Mississippi; NC, North Carolina.

| **Table S3.** **ARIC community coronary heart disease surveillance**  **decedent characteristics of records listing heart failure as the underlying cause of death^a^** | |
| --- | --- |
| **Characteristic** | **Weighted N (%)** |
| Sex |  |
| Male | 348 (52.8) |
| Female | 312 (47.2) |
| Race |  |
| White | 417 (63.3) |
| Black/African American | 236 (35.9) |
| Asian | 4 (0.7) |
| American Indian/Native Alaskan | 1 (0.2) |
| Center |  |
| Forsyth County, NC | 227 (34.5) |
| Jackson, MS | 262 (39.7) |
| Minneapolis, MN | 104 (15.7) |
| Washington County, MD | 67 (10.2) |
| Place of Death |  |
| In-Hospital | 235 (35.6) |
| Out of Hospital | 425 (64.4) |
| Year of Death |  |
| 1999 | 35 (5.3) |
| 2000 | 37 (5.7) |
| 2001 | 29 (4.4) |
| 2002 | 42 (6.4) |
| 2003 | 33 (5.0) |
| 2004 | 29 (4.4) |
| 2005 | 108 (16.5) |
| 2006 | 82 (12.4) |
| 2007 | 87 (13.1) |
| 2008 | 75 (11.4) |
| 2009 | 48 (7.3) |
| 2010 | 53 (8.1) |
| ^a^ Decedents age >=55, years 1999-2010; Weighted N=660; Note: the numbers were rounded down | |

| **Table S4.** **ARIC community surveillance coronary heart disease classification of records listing heart failure as the underlying cause of death, decedents age >=55, years 1999-2010, weighted N=660** | | | | | | | | | |
| --- | --- | --- | --- | --- | --- | --- | --- | --- | --- |
| **Classification** | **Total** |  | **Male** |  | **Female** |  | **Caucasian^a^** |  | **African American^a^** |
|  | **Weighted**  **N (%)** |  | **Weighted**  **N (%)** |  | **Weighted**  **N (%)** |  | **Weighted**  **N (%)** |  | **Weighted**  **N (%)** |
| ARIC classification of CHD based on an algorithm |  |  |  |  |  |  |  |  |  |
| CHD death  (definite MI, definite CHD and probable CHD) | 147 (22.3) |  | 86 (24.7) |  | 61 (19.6) |  | 94 (22.4) |  | 53 (22.6) |
| Non-CHD death | 416 (63.1) |  | 217 (62.4) |  | 199 (63.9) |  | 272 (65.2) |  | 139 (58.7) |
| Unclassifiable | 96 (14.6) |  | 45 (12.8) |  | 51 (16.5) |  | 52 (12.4) |  | 44 (18.7) |
| ARIC classification of CHD based on reviewer’s preferred classification ^b^ |  |  |  |  |  |  |  |  |  |
| CHD death  (definite MI, definite CHD and probable CHD) | 145 (27.5) |  | 89 (31.5) |  | 56 (22.8) |  | 94 (29.8) |  | 50 (24.7) |
| Non-CHD death | 221 (41.9) |  | 111 (39.3) |  | 110 (45.0) |  | 147 (46.3) |  | 73 (35.7) |
| Unclassifiable | 61 (11.7) |  | 27 (9.4) |  | 35 (14.3) |  | 31 (9.9) |  | 30 (14.8) |
| Discrepancy between reviewers | 100 (18.9) |  | 56 (19.8) |  | 44 (17.9) |  | 45 (14.1) |  | 51 (24.9) |
| Abbreviations: CHD, coronary heart disease; MI, myocardial infarction  ^a^ excluding other race  ^b^ 86 records did not get reviewed and were not included in the weighted N  Note: the numbers were rounded down | | | | | | | | | |

| **Table S5. ARIC community surveillance coronary heart disease classification of records listing heart failure as the underlying cause of death by field center, decedents age >=55, years 1999-2010, weighted N=660** | | | | | | | |
| --- | --- | --- | --- | --- | --- | --- | --- |
| **Classification** | **Forsyth County, NC** |  | **Jackson, MS** |  | **Minneapolis, MN** |  | **Washington County, MD** |
|  | **Weighted**  **N (%)** |  | **Weighted**  **N (%)** |  | **Weighted**  **N (%)** |  | **Weighted**  **N (%)** |
| ARIC classification of CHD based on an algorithm |  |  |  |  |  |  |  |
| CHD death (definite MI, definite CHD  and probable CHD) | 51 (22.5) |  | 74 (28.1) |  | 16 (15.2) |  | 6 (9.6) |
| Non-CHD death | 152 (67.0) |  | 133 (50.8) |  | 78 (74.9) |  | 54 (80.1) |
| Unclassifiable | 24 (10.5) |  | 55 (21.0) |  | 10 (9.9) |  | 7 (10.3) |
| ARIC classification of CHD based on reviewer’s preferred classification ^a^ |  |  |  |  |  |  |  |
| CHD death (definite MI, definite CHD  and probable CHD) | 48 (29.6) |  | 75 (31.3) |  | 13 (17.4) |  | 9 (17.3) |
| Non-CHD death | 74 (46.1) |  | 83 (34.4) |  | 36 (49.1) |  | 28 (53.8) |
| Unclassifiable | 15 (9.1) |  | 35 (14.4) |  | 9 (12.0) |  | 3 (6.6) |
| Discrepancy between reviewers | 25 (15.2) |  | 48 (19.9) |  | 16 (21.6) |  | 12 (22.3) |
| Abbreviations: CHD, coronary heart disease; MI, myocardial infarction  ^a^ 86 records did not get reviewed and were not included in the weighted N  Note: the numbers were rounded down | | | | | | | |
